# *APOE4* to *APOE2* ‘Switching’ in Astrocytes Alters the Cerebral Transcriptome and Decreases AD Neuropathology at Different Points on the AD Pathophysiological Timeline

**DOI:** 10.1002/alz.090743

**Published:** 2025-01-03

**Authors:** Lesley R Golden, Isaiah O Stephens, Dahlia Siano, Steven M MacLean, Kumar Pandya, Scott Gordon, Josh M. Morganti, Lance A Johnson

**Affiliations:** ^1^ Sanders‐Brown Center on Aging, Lexington, KY USA; ^2^ University of Kentucky, Lexington, KY USA; ^3^ University of North Carolina Chapel Hill, Chapel Hill, NC USA; ^4^ Saha Cardivascular Research Center, Lexington, KY USA; ^5^ University of Kentucky College of Medicine, Sanders‐Brown Center on Aging, Lexington, KY USA

## Abstract

**Background:**

Compared to the ‘neutral’ E3, the E4 allele of Apolipoprotein E (*APOE*) confers up to a 15‐fold increase in Alzheimer’s Disease (AD) risk. Conversely, the neuroprotective E2 allele decreases AD risk by a similar degree. Here, we aimed to assess the therapeutic potential of cell‐type specific allelic ‘switching’ by investigating the physiological and neuropathological changes associated with an inducible, *in vivo APOE4* to *APOE2* transition in astrocytes using a novel transgenic mouse model

**Method:**

The *APOE* “switch mouse” (APOE4s2) uses the Cre‐loxP system to allow for inducible *APOE* allele switching from E4 to E2. These mice express a floxed human *APOE4* coding region followed by the human *APOE2* coding region. Allelic discrimination (RT‐PCR) and mass spec‐based proteomic analyses were employed to validate the E4 to E2 transition. Single‐cell RNAseq and Xenium In Situ were used to measure transcriptomic changes following the astrocytic E4 to E2 allele switch. Behavioral measures and neuropathological analyses were applied to assess the effects of an early‐life vs. mid‐life allelic switch on AD pathology.

**Result:**

mRNA and protein analyses confirm that APOE4s2 mice synthesize full‐length human *APOE4* pre‐switch, and that tamoxifen induces an efficient recombination and expression of human *APOE2* in target tissues. Single‐cell RNAseq and Xenium reveal that, genetic replacement of astrocytic *APOE4* with *APOE2* results in distinct alterations to glial cell transcriptomes affecting pathways involved with metabolism, inflammation, and amyloid beta. Neuropathological analyses show that an astrocyte‐specific E4 to E2 ‘switch’ significantly decreases total amyloid burden, even once severe amyloidosis has already begun (mid‐life ‘switch’), and that the astrocytic E4 to E2 transition improves cognition. Additionally, amyloid‐associated astro‐ and micro‐gliosis are decreased, and mice with E2‐expressing astrocytes had less MHCII expressing microglia and a decrease in plaque‐associated ApoE.

**Conclusion:**

Together, these data suggest that a successful transition from E4 to E2 in astrocytes has broad impact on the cerebral transcriptome and that an astrocyte‐specific E4 to E2 ‘switch’ improves multiple AD‐associated pathologies at different points across the AD pathophysiological timeline.

